# Neuropsychiatric Systemic Lupus Erythematosus: A Systematic Review

**DOI:** 10.7759/cureus.61678

**Published:** 2024-06-04

**Authors:** Tyler E Rice-Canetto, Sonali J Joshi, Katie A Kyan, Javed Siddiqi

**Affiliations:** 1 Neurosurgery, California University of Science and Medicine, Colton, USA; 2 Neurosurgery, Arrowhead Regional Medical Center, Colton, USA; 3 Neurosurgery, Desert Regional Medical Center, Palm Springs, USA; 4 Neurosurgery, Riverside University Health System Medical Center, Moreno Valley, USA

**Keywords:** cognitive dysfunction, status epilepticus (se), transverse myelitis, auto immune, neurology and psychiatric disorders, american college of rheumatology (acr), systemic lupus erythematosus, neuropsychiatric systemic lupus erythematosus (npsle)

## Abstract

Neuropsychiatric systemic lupus erythematosus (NPSLE) refers to the neurological and psychiatric manifestations of systemic lupus erythematosus (SLE), which remain poorly understood yet often have a profound effect on the lives of afflicted patients. The aim of this study is to synthesize the available information on the pathogenesis, diagnostics, management, and prognosis of this disease. Our hope is to increase awareness and call for further investigations that may optimize NPSLE patient outcomes and quality of life. We performed a literature review following the Preferred Reporting Items for Systematic reviews and Meta-Analyses guidelines, resulting in 11 studies of inclusion. Within each study, we extracted data on epidemiologic factors, diagnostics, therapeutic modalities, and prognosis for each neuropsychiatric condition. The most widely discussed neuropsychiatric manifestations of SLE based on the American College of Rheumatology (ACR) classifications included status epilepticus (SE) and seizures, transverse myelitis (TM), and cognitive dysfunction. SE and TM had a prevalence of 1-2%, while cognitive dysfunction was nearly 38%. Diagnostics varied depending on symptom presentation but often included brain magnetic resonance imaging (MRI) and antibody testing. Treatment for NPSLE is still widely understudied, but concurrent treatment with immunosuppressants and anti-inflammatories for symptom control and more targeted immunotherapies based on the specific condition is often effective. Prognosis is highly symptom dependent, ranging from a 12.5% one-year mortality in SE and seizure patients to near resolution of symptoms in certain presentations including idiopathic intracranial hypertension and cerebellar ataxia. Further studies are needed to better understand the pathophysiology, diagnostics, and effective therapeutic measures for NPSLE. The severity of these manifestations and generally poor prognosis highlight the need for more research to accurately diagnose and treat this disease. While there is still little data available, this literature review serves to provide updated context on this condition.

## Introduction and background

The autoimmune disorder systemic lupus erythematosus (SLE) is one of the most common types of lupus in which the immune system attacks its own tissues [1]. SLE can cause inflammation and damage to almost every organ system in the body including the brain, skin, joints, and lungs. The prevalence of SLE is higher among women compared to men, with approximately six women affected for every male [1]. Although there have been many advancements in therapeutics and diagnostic measures for SLE, the exact pathogenesis of the disease is unknown, and it remains a major contributor to morbidity and mortality around the globe [1]. One significant manifestation of SLE is nervous system involvement, termed neuropsychiatric systemic lupus erythematosus (NPSLE). It can affect both the central and peripheral nervous systems (PNS) in a number of ways. According to the 1999 American College of Rheumatology (ACR) guidelines, there are 19 conditions that can be observed in NPSLE, including headaches, seizure disorders, and many others [2]. The ACR further classifies these conditions as affecting the central nervous system (CNS) or the PNS. In addition to these 19 syndromes, six additional neuropsychiatric manifestations of SLE are prevalent in the literature and will be addressed in this paper. The pathophysiology of NPSLE is not fully understood, but it is speculated that it could be as a result of certain antibodies [3,4]. Other theories include that the NPSLE symptoms occur as a consequence of SLE treatments. The European League Against Rheumatism (EULAR) released guidelines in 2010 surrounding the treatment of NPSLE including the management of the 19 ACR syndromes and these additional manifestations [5,6]. A table detailing the breakdown of the 19 ACR conditions as central or peripheral manifestations, and the additional six manifestations, may be found in Table 1.

**Table 1:** American College of Rheumatology NPSLE criteria and additional categorizations ACR: American College of Rheumatology; NPSLE: neuropsychiatric systemic lupus erythematosus

ACR Central Nervous System Manifestations-Neurological	ACR Central Nervous System Manifestations-Psychiatric	ACR Peripheral Nervous System Manifestations	Non-ACR Manifestations
Acute confusional state	Anxiety disorder	Acute inflammatory demyelinating polyradiculopathy (Guillain-Barré)	Cerebellar ataxia
Aseptic meningitis	Mood disorder	Autonomic disorder	Cerebral venous sinus thrombosis
Cerebrovascular disease	Psychosis	Cranial neuropathy	Idiopathic intracranial hypertension
Cognitive dysfunction		Myasthenia gravis	Isolated optic neuritis
Demyelinating syndrome		Plexopathy	Posterior reversible encephalopathy syndrome
Headache		Polyneuropathy	Progressive multifocal leukoencephalopathy
Movement disorder		Simple/multiple mononeuritis	
Myelopathy			
Seizure disorder			

Of the 19 ACR syndromes, three were predominantly discussed in the literature. These three include status epilepticus (SE) and seizures, transverse myelitis (TM), and cognitive dysfunction. Each of these manifestations have varying prevalences, treatments, and prognoses. Understanding how these conditions present clinically and intertwine with the diagnosis of SLE is crucial in supporting patients with this condition. This literature review aims to provide updated information on NPSLE in efforts to improve diagnostics and prognosis for affected patients.

## Review

Methods

This systematic review was conducted based on recommendations put forth by the Preferred Reporting System for Systematic Reviews and Meta-Analyses (PRISMA) statement protocol.

Data Sources and Search Strategies

To conduct our systematic review of the literature on the neurological and psychiatric manifestations of SLE, we thoroughly searched the databases PubMed and Embase. For our PubMed search, we included articles from the induction of the database in January of 1996 through January 25, 2024. For our Embase search we included articles published between the dates of January 1, 2021, and January 25, 2024, given that the most recent literature review on NPSLE to date was published in January of 2021 with references from Embase and MEDLINE. For our search in Embase, we used the following filters in addition to date: Embase and MEDLINE publications, human subjects, publication type of “article in press” or “review,” and the language English. We completed an advanced search on both the PubMed and Embase database sites, and our terms included the following: ("Neuropsychiatric lupus" OR "Neuropsychiatric systemic lupus erythematosus" OR "NPSLE" OR "NP systemic lupus erythematosus" OR "Neurological Lupus" OR "Neurologic systemic lupus erythematosus" OR "Neurologic SLE" OR "Psychiatric lupus" OR "Psychiatric systemic lupus erythematosus" OR "Psychiatric SLE") AND ("manifestation" OR "complication" OR "sequelae" OR "diagnosis" OR "detection" OR "screening" OR "assessment" OR "evaluation" OR "identification" OR "characterization" OR "classification" OR "characteristics" OR "clinical features" OR "symptoms" OR "signs" OR "prognosis" OR "outcome" OR "survival" OR "prognostic factors" OR "prognostic markers" OR "treatment" OR "therapeutics" OR "manifestations" OR "management" OR "interventions" OR "therapy" OR "diagnostic criteria" OR "diagnostic algorithm" OR "algorithm" OR "prevention").

Inclusion and Exclusion Criteria

Our review includes publications that specifically address one or multiple of the 19 neurologic or psychiatric manifestations of SLE that are outlined by the 1999 ACR guidelines. We also included any papers focusing on one or multiple of the well-documented neuropsychiatric manifestations of lupus since the ACR released their guidelines, including cerebral venous sinus thrombosis (CVST), posterior reversible encephalopathy syndrome (PRES), isolated optic neuritis, progressive multifocal leukoencephalopathy (PML), idiopathic intracranial hypertension, and cerebellar ataxia [5,7]. Our eligibility criteria included case reports, literature reviews, systematic reviews, and meta-analyses published in English. We did not include papers whose primary focus did not meet one of the aforementioned conditions, in addition to any of the following specific exclusion criteria: papers limited to a specific age group of pediatric or elderly, clinical trials examining novel SLE treatments with minimal discussion of NPSLE specifically, animal and laboratory studies, publications requiring payment for access, and publications which are not scientifically published such as editorials and opinion columns. 

Data Extraction

Each study was independently screened and reviewed by three reviewers (TR, KK, SJ) for eligibility based on the abovementioned inclusion and exclusion criteria. Each reviewer was then assigned to a number of publications to extract clinical data, including study design, age at diagnosis, sex, general SLE symptoms, prevalence of NPSLE in a given cohort, stage of presentation of neuropsychiatric condition, counts and relevant information from each cohort on the 19 ACR conditions or the additional included five specific manifestations, diagnostic modalities and findings, biomarkers, therapeutics and disease-modifying agents, and prognosis. 

Each publication’s extractions were then independently reviewed by one of the other two authors. In the case that there was a discrepancy in the data, reviewers discussed and came to a unanimous agreement before proceeding.

Search of the Literature 

**Figure 1:**
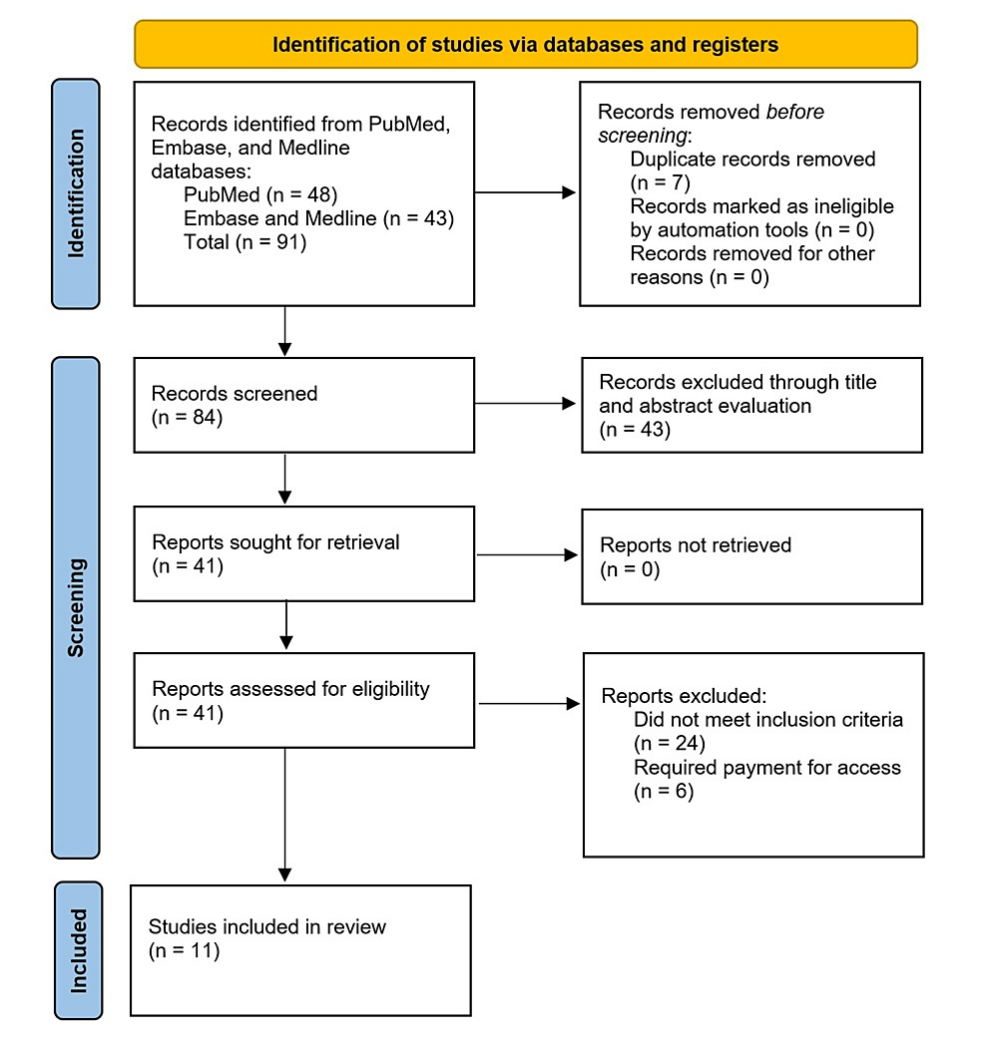
Flowchart of publication selection process based on PRISMA guidelines PRISMA: Preferred Reporting Items for Systematic Reviews and Meta-Analyses

On January 25, 2024, a search on PubMed and Embase with the previously mentioned dates and filters resulted in a total of 91 potentially eligible articles, 48 from PubMed and 43 from a combination of Embase and MEDLINE via the Embase database search function. After removal of duplicate publications, 84 articles remained. By screening abstracts and publication titles to eliminate papers not specifically addressing the neuropsychiatric manifestations of SLE, we were left with 41 publications. With a more comprehensive review of said articles, 30 records were eliminated, 24 of which did not meet our inclusion criteria and six of which we were unable to freely access via our institution. Finally, 11 studies were selected for inclusion and clinical data was extracted by our three authors (TR, KK, SJ). Figure 1 demonstrates a flowchart of the article screening process of this review paper. Table 2 includes details of each paper included in our review.

**Table 2:** Final papers included in review based on PRISMA diagram ANA: Antinuclear; ANAM: automated neuropsychological assessment metrics; aPL: antiphospholipid; APRIL: a proliferation-inducing ligand; BAFF: B cell activating factor; CCL2/MCP-1: chemokine (CC-motif) ligand 2/monocyte chemoattractant protein-1; COWAT: controlled oral word association test; CSF: cerebrospinal fluid; CT: computed tomography; CXCL10: C-X-C motif chemokine ligand 10; dsDNA: double-stranded DNA; EEG: electroencephalogram; EMG: electromyography; fMRI: functional magnetic resonance imaging; GADPH: glyceraldehyde-3-phosphate dehydrogenase; HVLT-R: Hopkins verbal learning test; IgG: immunoglobulin G; IFN: interferon; IL: interleukin; LP: lumbar puncture; MMSE: mini-mental status exam; MoCA: Montreal cognitive assessment; MRA: magnetic resonance angiography; MRI: magnetic resonance imaging; NA: not applicable; NMDAR: N-methyl-D-aspartate receptor; NR2: anti-N-methyl-d-aspartate receptor subunit NR2; PMID: PubMed identifier; PRISMA: Preferred reporting Items for Systematic Reviews and Meta-Analyses; RNP: ribonucleoprotein; SSA and SSB: Sjögren’s syndrome A and B; TNF: tumor necrosis factor; TWEAK: TNF-related weak inducer of apoptosis

Author and Publication Year	Article Title	PMID	Study Design	Age	Sex	Diagnostic Methods	Biomarkers	Therapeutics
Khan et al., 2023 [1]	Prevalence of neuropsychiatric disorders in patients with systemic lupus erythematosus in Pakistan: a systematic review and meta-analysis	36816415	Systematic review and meta-analysis	29.86 years average	76-96% female	NA	NA	NA
Zhang et al., 2020 [2]	Neuropsychiatric lupus erythematosus: future directions and challenges; a systematic review and survey	32321114	Systematic review and survey	32.1 +/- 12.1 years average	NA	CSF analysis, EEG, neurophysical examination, biopsy, neuroimaging	Anti-APRIL antibodies, TNF, IL-6, IL-1, BAFF, IFN-α, aPL antibodies, alpha-tubulin, NMDAR antibodies, GADPH antibodies	Methotrexate + dexamethasone, rituximab, hematopoietic stem cell transplant, antiepileptics, anticoagulation
Carrión-Barberà et al., 2021 [5]	Neuropsychiatric involvement in systemic lupus erythematosus: a review	33609799	Systematic review	NA	Female predominance	LP, brain biopsy, cardiovascular reflex assessment, electrophysical studies, nerve biopsy, EMG	aPL antibodies, AQP4 antibodies, anti-ribosomal P antibodies, NMDAR antibodies, IL-6, anti-NR2 antibodies, anti-Smith antibodies, acetylcholine receptor antibodies	Glucocorticoids, cyclophosphamide, immunoglobulins, immunosuppressants, antiepileptics, anti-dopaminergics, plasmapheresis, haloperidol, atypical antipsychotics, rituximab, aspirin
Sy et al., 2021 [7]	Cerebellar ataxia as a primary manifestation of neuropsychiatric systemic lupus erythematosus	33542005	Literature review	14-41 years range	Female predominance	CT head, EEG, CSF studies, MRI brain, PET brain, EMG and nerve conduction studies	ANA, anti-dsDNA antibodies, anti-SSA antibodies, anti-Smith antibodies, anti-SSB antibodies, anti-Ro ribonucleoprotein antibodies	Immunosuppressants, azathioprine, cyclophosphamide, hydroxychloroquine
Mikdashi et al., 2023 [8]	Long-term outcome of status epilepticus-related to systemic lupus erythematosus: an observational study and a systematic review	37595509	Observational study and systematic review	31.6 +/- 10.3 years average	62.5% female, 37.5% male	Neurological exam, metabolic panel, toxicology screen, CSF studies, CT head, MRI/MRA brain, EEG	Anti-dsDNA antibodies, aPL antibodies, anti-SSA antibodies	Cyclophosphamide, prednisone, phenytoin, antiepileptics, benzodiazepines, ketamine, pregabalin, methylprednisone, immunoglobulins, immunosuppressants, rituximab
Rodriguez-Hernandez et al., 2021 [9]	Seizures in systemic lupus erythematosus: a scoping review	33626435	Literature review	22.9-36.5 years range	Female predominance	CSF analysis	IL-6, IL-8, IL-17. IgG ANA, aPL antibodies, anti-Ro ribonucleoprotein antibodies, anti-B2 glycoprotein antibodies	Immunosuppressants, antiepileptics, glucocorticoids, cyclophosphamide, hydroxychloroquine, aspirin
Wang et al., 2022 [10]	Relapse rates and risk factors for unfavorable neurological prognosis of transverse myelitis in systemic lupus erythematosus: a systematic review and meta-analysis	34798313	Systematic review and meta-analysis	NA	88.7-96.5% female	Meeting diagnostic criteria for transverse myelitis: sensory/motor/autonomic dysfunction + bilateral symptoms + exclusion of extra-axial compressive etiology via neuroimaging + spinal cord inflammation demonstrated by CSF pleocytosis + elevated IgG enhancement/gadolinium enhancement + progression to nadir between 4 and 21 days after symptom onset	aPL antibodies, anticardiolipin antibodies, anti-B2 glycoprotein antibodies, lupus anticoagulant	Glucocorticoids + cyclophosphamide
Rayes et al., 2018 [11]	What is the prevalence of cognitive impairment in lupus and which instruments are used to measure it? A systematic review and meta-analysis	29571540	Systematic review and meta-analysis	NA	NA	Cognitive battery tests, complex attention tests, memory and learning recall tests, visual-spatial processing tests, language tests, reasoning/problem solving tests, ANAM, MMSE, MoCA, HVLT-R, COWAT	aPL antibodies	NA
Mizrachi et al., 2022 [12]	Cognitive dysfunction in SLE: an understudied clinical manifestation	36127204	Literature review	NA	NA	Neuropsychological batteries, neuroimaging	Type 1 IFN, IL-6. TNF, TWEAK, CXCL10, CCL2/MCP-1	Immunosuppressants, cyclophosphamide, rituximab, antiepileptics, antipsychotics
Mikdashi, 2016 [13]	Altered functional neuronal activity in neuropsychiatric lupus: a systematic review of the fMRI investigations	26897255	Systematic review	37.6 years average for adults, 15.7 years average for children	9:1 female:male ratio	fMRI	NA	NA
Meier et al., 2021 [14]	Neuro-psychiatric manifestations in patients with systemic lupus erythematosus: a systematic review and results from the Swiss lupus cohort study	34152246	Systematic review and cohort study	44.3 years average	89.6% female, 10.4% male	NA	ANA, anti-dsDNA antibodies, anti-Sm antibodies, anti-SSA antibodies, anti-U1-RNP antibodies	NA

Paper overview

In completing our literature review, the most widely discussed neuropsychiatric manifestations of SLE included SE and seizures, TM, and cognitive dysfunction. For each of these ACR neuropsychiatric conditions, we will go on to discuss in depth epidemiology, symptomatology, method of diagnosis and biomarkers, therapeutic management, and prognosis. For the remainder of the 19 ACR NPSLE conditions, of which a complete list may be referenced in Table 1, we will more generally summarize the clinical presentation, diagnostics, treatment, and patient outcomes. Additionally, we will address six conditions that are not included in the 19 ACR diagnostic criteria but are discussed abundantly in the literature on NPSLE. These include CVST, PRES, isolated optic neuritis, PML, idiopathic intracranial hypertension, and cerebellar ataxia.

SE and Seizures

SE is a prolonged seizure disorder that can manifest as a complication in about 1-2% of patients with SLE [8]. The publication “Long-term outcome of status epilepticus-related to systemic lupus erythematosus: An observational study and a systematic review” by Mikdashi and Krumholz provides an overview of SE-related to lupus (SE-SLE) and its characteristics, neuroimaging studies, pathophysiology, and clinical outcomes [8]. The observational study consisted of 40 SE-SLE patients and included cases identified from a single tertiary care hospital and subsequent systematic review. Common symptoms of SE-SLE were revealed with an analysis of the reported cases which showed that most patients presented with new-onset seizures (82.5%) compared to prior generalized epilepsy. Types of SE-SLE varied from patients who presented with prolonged episodes of sensory symptoms known as aura continua to patients in a coma state, identified as the slowing of background activity on EEG or epileptiform discharges showing rhythmic slow activities. Patients were further classified as having either convulsive or nonconvulsive SE-SLE. Diagnostically, almost half of SE-SLE patients showed epileptiform discharges on EEG. The authors utilized the International League Against Epilepsy (ILAE) criteria to assign the etiology of SE-SLE into acute symptomatic, progressive symptomatic, remote symptomatic, and cryptogenic/idiopathic causes. The etiology of these symptoms was mainly found to be related to autoimmunity rather than cerebrovascular disease or cryptogenic causes [8]. Another publication by Rodriguez-Hernandez et al. further supports this theory, stating that inflammatory cells and antibodies have a large role in the pathogenesis of seizures in SLE and that understanding these mechanisms is key to developing targeted therapies for such patients [9]. 

The authors Mikdashi et al. diagnosed SE-SLE using criteria from the International Classification of Epileptic Seizures. It is defined as a seizure event lasting five minutes or longer or as continuous clinical and/or EEG seizure activity or recurrent activity without recovery between episodes [8]. Diagnostic tools included metabolic panels, CSF studies, brain MRI/MR angiography, and extended EEG monitoring. SE-SLE findings on imaging commonly showed focal vasculitic lesions associated with cognitive impairment and less commonly seen were reversible MRI abnormalities, cerebral atrophy, and sclerosis. However, the authors concluded that diagnosis of SE-SLE was not associated with any specific imaging results or laboratory abnormalities, including biomarkers [8]. 

Therapeutic management guidelines for SE-SLE included intravenous benzodiazepines with follow-up treatment using a longer acting anti-seizure medication. Intravenous high-dose methylprednisolone was used in all SE-SLE patients, and many patients also required supplementary aggressive immunosuppressive therapies. Follow-up of patients in this observational study ultimately found that long-term outcomes of SE-SLE are poor with a one-year mortality rate of 12.5%, significant in-hospital deaths, recurrent SE-SLE/epilepsy, cognitive impairment, and limited physical function [8]. 

TM

TM is another manifestation of NPSLE that occurs in 1-2% of SLE patients and involves symptoms of paralysis, sensory deficits, and sphincter dysfunction [10]. In a publication titled “Relapse rates and risk factors for unfavorable neurological prognosis of transverse myelitis in systemic lupus erythematosus: A systematic review and meta-analysis,” Wang et al. describes the factors affecting prognosis for SLE-related TM [10]. The study included 283 SLE-associated TM cases and all patients who met the criteria for diagnosis of myelitis such as the development of sensory, motor, or autonomic spinal cord dysfunction and CSF pleocytosis showing inflammation within the spinal cord [10].

Upon analysis of clinical characteristics in SLE-related TM, no association was found between age of onset and poor outcome. However, the authors used the American Spinal Injury Association Impairment Scale (AIS) to assess neurological outcomes in these patients and determined that a grade of A, B, or C on the scale was strongly associated with poor prognosis. Similarly, a low glucose level in the CSF was also found to be related to a worse prognosis in SLE-related TM patients. No correlation was found between positive aPLs or different aPLs profiles and poor outcomes. Rather, extensive vasculitis throughout the spinal cord was a key marker in development of the disease. The authors suggest that neurological outcomes may be significantly improved by anti-inflammatory therapy [8].

Despite initial studies showing that methylprednisolone pulse treatments with cyclophosphamide are recommended for NPSLE, the authors found that neither treatment was associated with improved neurological outcomes. Instead, they suggest that treatment plans should be stratified based on the initial severity of TM [8]. Patients with poor prognosis indicated by an AIS grade of A, B, or C are recommended to be given rituximab, plasma exchange, or anti-IL-6 agents as soon as possible in the case of severe attacks. Relapse rates were high with recurrence of TM occurring at least once in 21-55% of patients. Recurrence rates were higher in the first year after the initial event and stabilized in 3-5 years which may signify the importance of early treatment [10].

Cognitive Dysfunction

Cognitive dysfunction is another of the most widely discussed conditions of NPSLE. In a publication titled “What is the prevalence of cognitive impairment in lupus and which instruments are used to measure it? A systematic review and meta-analysis,” Rayes et al. discussed the prevalence of cognitive impairment in those with SLE [11]. They found that the pooled prevalence of cognitive dysfunction in 2,463 SLE patients tested by comprehensive battery (CB) was 38% [11].

The methods of diagnosing cognitive dysfunction in patients with SLE are an important factor in understanding this presentation. CB tests are most frequently used to test factors such as simple/complex attention, memory/learning recall, visual-spatial processing, language, reasoning/problem-solving, and processing speed. Another frequently used test is the Automated Neuropsychological Assessment Metric (ANAM), a self-administered computerized cognitive test battery. Other diagnostic tests that are less utilized include the mini-mental state exam (MMSE), Montreal cognitive assessment (MoCA), controlled oral word association Test (COWAT), and the Hopkins verbal learning test-revised (HVLT-R) [11].

Brain imaging is also crucial for diagnosing cognitive dysfunction in those with SLE. These include typical neuroimaging tools including MRI, fMRI, PET, and dynamic MRI [12,13]. Imaging of patients with cognitive dysfunction will reveal decreased brain matter volumes in the hippocampus and amygdala, decreased white matter integrity in numerous regions, and increased blood-brain barrier permeability in the temporal and occipital lobes, cerebellum, and brainstem. Studies will also show decreased perfusion in frontal, temporal, parietal lobes, and basal ganglia and numerous changes in brain function including decreased hippocampal network activity and changes in metabolism onset [12].

Certain biomarkers are also present in those with cognitive dysfunction such as type 1 interferon (IFN), interleukin-6 (IL-6), tumor necrosis factor (TNF), TNF-related weak inducer of apoptosis (TWEAK), C-X-C motif chemokine ligand 10 (CXCL10 (IP-10)), and monocyte chemoattractant protein-1 (CCL2/MCP-1). One important biomarker which also serves as an indication of disease prognosis is antiphospholipid antibodies. Despite the availability of information on diagnostics, there is still much to be elucidated in terms of treatment for NPSLE patients with the specific presentation of cognitive dysfunction.

The remainder of ACR NPSLE manifestations

Though the aforementioned three conditions, SE and seizures, TM, and cognitive dysfunction are the most widely discussed manifestations of NPSLE, the other 16 conditions discussed by the ACR are equally important. Presentations such as headaches and mood disorders are seen most commonly, while others such as demyelinating disease and aseptic meningitis are rare [5,14]. When considering how to treat and diagnose these conditions, most are thought of as individual presentations, rather than being treated as a part of the patient’s SLE. For example, if a patient presented with headaches, the headache would be addressed independently, rather than as a SLE-associated headache. The most common treatment option for most symptoms is immunosuppression, typically with cyclophosphamide [15,16]. As more information is revealed about NPSLE, information about these important conditions can be elucidated as well.

Additional non-ACR NPSLE manifestations

In addition to the 19 categories of CNS and PNS conditions as defined by the ACR, there are five well-documented neuropsychiatric manifestations of SLE including CVST, PRES, isolated optic neuritis, PML, and idiopathic intracranial hypertension. Each of these conditions occurs in less than 1% of SLE patients. The symptomatology, diagnostics, management, and prognosis of each of these presentations is well described in a publication entitled “Neuropsychiatric involvement in systemic lupus erythematosus: A review” by Carrión-Barberà et al. [5]. We will further outline each of these clinical presentations of NPSLE. Additionally, we will detail one more manifestation not included in the ACR guidelines, which is cerebellar ataxia. That discussion will be based on the publication entitled “Cerebellar ataxia as a primary manifestation of neuropsychiatric systemic lupus erythematosus” by Sy et al. [7].

CVST refers to the formation of a blood clot in the brain’s venous sinuses, resulting in a blockage of venous blood drainage from the brain. This can potentially cause swelling of the brain tissue and elevated intracranial pressure (ICP). In the case of SLE patients, the most commonly affected anatomical site is the transverse sinus. CVST most commonly presents as signs and symptoms of elevated ICP which may include headache, nausea and vomiting, seizures, visual disturbances, or altered mental status. For diagnostics, angiography or computed tomography angiography (CTA) of the venous system are most commonly used. Treatment is the same as for non-SLE patients with CVST and includes anticoagulation with heparin followed by vitamin K anticoagulants such as warfarin. For patients with NPSLE, treatment with a combination of glucocorticoids and immunosuppressants has shown clinical efficacy [5].

The pathophysiology of PRES remains unclear; however, one proposed hypothesis is that its mechanism is based on the brain’s inability to properly autoregulate and maintain consistent cerebral blood flow over a range of blood pressures. The body increases vasoconstriction as blood pressures elevate in order to maintain perfusion of the brain tissue, leading to increased blood flow and thus elevated hydrostatic pressures within the brain. A consequence of this is increased breakdown of the blood-brain barrier and subsequent edema. Similar to CVST, symptoms include headache, seizures, visual impairment, altered mentation, and drowsiness [17]. Diagnostically, MRI brain will show bilateral vasogenic edema, more specifically within the posterior circulation. In NPSLE patients, management is with blood pressure control and a combination of glucocorticoids and immunosuppressants. PRES has an overall favorable prognosis with SLE patients. However, even in patients already taking glucocorticoids and immunosuppression, there are reported cases of PRES. More research is needed on the topic to determine the most effective treatment regimen [5]. 

Optic neuritis in the setting of SLE is most often the consequence of demyelination and axonal necrosis secondary to ischemia, with the extent of necrosis dictating degree of visual impairment [5]. For the majority of patients with NPSLE, optic neuritis occurs in conjunction with TM, which is discussed in a prior section. “Isolated” optic neuritis is the focus of this discussion, referring specifically to the presentation of optic neuritis independent of TM. Clinical presentation includes reduced visual acuity with potential progression to blindness, central scotomas, and ocular pain. Optic neuritis is often diagnosed via ophthalmological exam, and in some cases may be associated with antiphospholipid syndrome (APS) antibodies [5]. SLE patients with isolated optic neuritis are often treated with high-dose corticosteroids, starting with three days of IV methylprednisolone. As an add-on therapy to reduce risk of thrombosis in blood vessels supplying the optic nerve, hydroxychloroquine may be used. Lastly, because of the association between isolated optic neuritis and APS, aspirin and vitamin K antagonists such as warfarin may be used in conjunction [5].

PML is a progressive and fatal demyelinating disorder that is a very rare condition of NPSLE. It is linked to the activation of the normally dormant JC virus in immunocompromised individuals, such as in SLE patients taking chronic immunosuppressants. Among the autoimmune disorders, PML is most commonly associated with SLE [5]. Chronic use of the immunosuppressant rituximab has been specifically associated with the reactivation of the JC virus. The symptomatic presentation of PML differs widely depending on which area of white matter has been demyelinated within the nervous system. Symptom presentation is diverse, ranging from cognitive impairment, to ataxia, hemiparesis, hemianopia, and aphasia [17,18]. MRI brain may reveal hyperintense regions in the T2 and FLAIR sequences, but a definitive diagnosis of PML is through brain biopsy. Management for NPSLE patients involves a balance between removing immunosuppressants that cause lymphopenia and avoiding exacerbation of SLE symptoms [5].

Idiopathic intracranial hypertension, also referred to as pseudotumor cerebri, describes a condition of elevated ICP without an identified etiology. Symptoms are based on those of an elevated ICP, namely, headache, nausea and vomiting, papilledema, and visual disturbances [5]. Diagnosis includes a combination of clinical presentations, elevated opening pressure on lumbar puncture, and normal brain imaging and CSF analysis findings. In some cases, it has been associated with anti-Ro antibodies [5]. Treatment involves glucocorticoids with the option to add IV acetazolamide or mannitol. In severe or refractory cases, VP shunt may be considered. Prognosis is generally good with proper adherence to medical management [5].

The final non-ACR neuropsychiatric manifestation of SLE to be discussed is cerebellar ataxia. Cerebellar ataxia refers to the loss of coordination of movements secondary to cerebellar damage, which in the setting of SLE is hypothesized to be secondary to cerebral ischemia, vasogenic edema, or antibody-mediated dysfunction [7]. This condition presents in <2% of SLE cases and may be diagnosed via imaging of the brain that reveals a cerebellar infarct, a solitary lesion at the pons-medulla junction, or general cerebellar atrophy [7]. Management includes high-dose corticosteroids, with the potential addition of immunomodulators such as hydroxychloroquine, azathioprine, or cyclophosphamide. Most patients see improvement of clinical symptoms after treatment [7].

Challenges

Neuropsychiatric lupus is a grossly understudied manifestation of SLE, despite the breadth and severity of symptoms it inflicts on many patients. The number of publications on the disease have increased over the last decade; however, there still remains little conclusive evidence regarding the most effective diagnostic approaches and therapeutic regimens for these patients. While there are established guidelines for what conditions may constitute NPSLE, there are no specific criteria that patients must meet to receive a formal diagnosis [19]. At this stage, the diagnosis is simply made in any SLE patient who presents with one or more of the defined neuropsychiatric manifestations as detailed in this paper [20]. Diagnostics become even more challenging in cases where patients have neuropsychiatric presentations that do not fall within the ACR guidelines, such as the supplementary diagnosis of cerebellar ataxia or isolated optic neuritis discussed in this paper. Even in instances where providers are able to identify neurological or psychiatric conditions in SLE patients, unfortunately these patients are rarely formally evaluated and therefore diagnosed or treated, as the battery of tests is resource intensive and time-consuming [14].

Because of the lack of awareness of NPSLE in conjunction with the prevalence of many of the conditions within the general population, many of these patients are going undiagnosed and thus untreated for their debilitating symptoms. It remains difficult to discern whether some of these patients' neuropsychiatric manifestations such as headache, anxiety, and depression are a consequence of their SLE or are independent, and how this differentiation affects management and outcomes [21]. Even in patients whose symptoms have been formally attributed to SLE, many treatments for these conditions are still trial and error at this stage, and further investigations and large scale clinical trials are necessary to optimize outcomes for these patients [22]. This review highlights the diverse clinical presentation and severity of symptoms in patients with NPSLE and calls for more research on the topic to optimize patient diagnostics and outcomes.

Review limitations

This review was limited by the small number of studies published on the topic, especially for those conditions not included in the ACR guidelines. Despite such limitations, this study serves to highlight the diverse symptom presentation that makes up neuropsychiatric lupus and the severity of manifestations that these patients may face. Further investigations are required to better understand the nuances of neuropsychiatric manifestations of SLE and how clinicians may more effectively identify and manage such symptoms [20,22]. 

## Conclusions

Symptomatic presentations of NPSLE span across various diseases including the proposed set of 19 syndromes classified by the ACR as well as other rare but well-documented manifestations. Each presentation differs in clinical characteristics, diagnostic measures, and other disease-defining traits, but general similarities include the importance of brain imaging and biomarkers as well as the balance between immunosuppressive and anti-inflammatory treatments. Although research on NPSLE presentations has increased over recent years, further studies are needed in order to better understand the pathophysiology, diagnostic criteria, and effective therapeutic measures for these conditions. The studies mentioned in this paper have contributed to our limited understanding of reliable lab markers/imaging findings, prognostic factors, and treatment options for NPSLE. This paper serves to refine the conclusions made by the papers included in our literature review, with the intention of enriching the existing body of literature that addresses this important subset of manifestations of SLE. The severity of NPSLE manifestations and generally poor prognosis highlights the need for more research that can help accurately diagnose and treat this disease.
